# Proximity to mining industry and respiratory diseases in children in a community in Northern Chile: A cross-sectional study

**DOI:** 10.1186/s12940-016-0149-5

**Published:** 2016-06-07

**Authors:** Ronald Herrera, Katja Radon, Ondine S. von Ehrenstein, Stella Cifuentes, Daniel Moraga Muñoz, Ursula Berger

**Affiliations:** Occupational and Environmental Epidemiology and NetTeaching Unit, Institute and Outpatient Clinic for Occupational, Social and Environmental Medicine, University Hospital Munich (LMU), Munich, Germany; Department of Community Health Sciences Fielding School of Public Health University of California Los Angeles, Los Angeles, CA USA; Facultad de Medicina, Universidad Diego Portales, Santiago, Chile; Facultad de Medicina, Latin American Center of the Center for International Health Ludwig-Maximilians-University, Universidad Católica del Norte, Coquimbo, Chile; Institute for Medical Informatics, Biometry and Epidemiology-IBE University of Munich (LMU), Munich, Germany

**Keywords:** Asthma, Children, Mining, Bayesian analysis, Air pollution

## Abstract

**Background:**

In a community in northern Chile, explosive procedures are used by two local industrial mines (gold, copper). We hypothesized that the prevalence of asthma and rhinoconjunctivitis in the community may be associated with air pollution emissions generated by the mines.

**Methods:**

A cross-sectional study of 288 children (aged 6–15 years) was conducted in a community in northern Chile using a validated questionnaire in 2009. The proximity between each child’s place of residence and the mines was assessed as indicator of exposure to mining related air pollutants. Logistic regression, semiparametric models and spatial Bayesian models with a parametric form for distance were used to calculate odds ratios and 95 % confidence intervals.

**Results:**

The prevalence of asthma and rhinoconjunctivitis was 24 and 34 %, respectively. For rhinoconjunctivitis, the odds ratio for average distance between both mines and child’s residence was 1.72 (95 % confidence interval 1.00, 3.04). The spatial Bayesian models suggested a considerable increase in the risk for respiratory diseases closer to the mines, and only beyond a minimum distance of more than 1800 m the health impact was considered to be negligible.

**Conclusion:**

The findings indicate that air pollution emissions related to industrial gold or copper mines mainly occurring in rural Chilean communities might increase the risk of respiratory diseases in children.

**Electronic supplementary material:**

The online version of this article (doi:10.1186/s12940-016-0149-5) contains supplementary material, which is available to authorized users.

## Background

The mining industry has been one of the most important contributors to the Chilean economy with a share of 13 % of the gross domestic product [1]. Gold and copper are extracted in open-pit mining facilities through excavation—a process that uses explosives and heavy machinery. Most of the air pollutants emerging form open pit mining are total suspended particulate (TSP) matter and particles, including aerodynamic diameter smaller than 10 μm (PM_10_) [2, 3]. The gold and copper mining activities generating these particles are mainly drilling, blasting, sediments loading and unloading, road transport over unpaved roads and losses from exposed over-burden dumps, residuals handling plants and exposed pit faces [4]. Dust after the explosion and transportation of materials and have been identified as the main source of TSP and PM_10_ pollution [5–7] in open-pit mining regions. High concentrations of particulate material (TSP and PM_10_) have been associated with asthma and allergies among others diseases [8–11]. Asthma is a common disease that affects children and adults of all ages. According to findings of the International Study of Asthma and Allergies in Childhood (ISAAC), the prevalence of asthma in childhood in Latin America is on average around 13 % and ranging between 13 and 16 % in Chile [12, 13].

Using measurements of air pollutants, associations between elevated levels of ambient air pollution and frequency of asthma attacks (emergency room visits/hospital admissions) have been shown [14–17]. Using proximity to the air pollution source as a proxy of exposure, studies suggested associations between living in areas close to industrial zones and the risk of asthma in children based on spatial analyses [18, 19]. In these studies, mainly logistic regression models and generalized additive models (GAM) [20] have been used. One limitation of the first approach is the use of arbitrarily chosen cut-off points for quartiles of exposure. For the latter, the interpretation of the results might be challenging, particularly when the association between the exposure and the outcome is non linear.

Countries in South America are experiencing high prevalence of asthma with rates ranging from 18 to 27 % [13, 21]. In Chile, higher prevalence of asthma and allergies was found in urban than in rural areas [22]. To the best of our knowledge associations between point sources of air pollution and respiratory health in children have not been studied in Chile to date. Exposure to open-pit mining is of particular interest as the exposure is widespread in Chile and other Latin American countries. In some communities, residents live less than one kilometer away from these open-pit mines.

Therefore, we examined associations between average distance of child’s place of residence to the two open-pit mines in a rural mining community and the prevalence of respiratory disease in children. The community is a mining dependent town located in Region IV in Chile with less than 10.000 inhabitants located at 1050 m above the sea level. Using data from a cross-sectional study, we assessed associations between the distance to the mines and respiratory diseases applying Bayesian semiparametric and parametric models including a spatial effect.

## Methods

### Study population

Data were used from a cross-sectional questionnaire based study in school children in a small town in Chile, which has one open-pit gold and one open-pit copper mine. Children and their parents or legal guardians provided written informed consent. The study included school children from 1st to 6th grade attending the two major local elementary schools. These two schools were the only ones in the community offering complete elementary education, and approximately 84 % of children in grades 1st to 6th were registered in these schools. Parental questionnaires with instructions were mailed to children’s homes. Further details about the study design are reported in Ohlander et al. [23].

### Data

The original study included 288 children (out of 418 children invited to participate, response rate: 69 %). Data on outcome variables and potential confounders were ascertained using a validated questionnaire in Spanish [24]. We groped the variables into three categories:

#### Child health outcomes

Asthma: a child was considered to have asthma, if the child was reported to ever have had asthma confirmed by a medical doctor or if the child had taken asthma medication during the last 12 months prior to the survey. Asthma medication was considered, because patients in Chile are often not aware of their asthma diagnosis (personal communication).

Rhinoconjunctivitis: positive report of one or more of the following nasal symptoms during the past 12 months prior to the survey: sneezing, itching, nasal congestion or rhinitis in conjunction with itchy, red, and watery eyes.

#### Characteristics of the participants

Sex (female vs. male), age (6–7 years vs. 8–9 years, 10–11 years and 12 years or more), parental history of atopic disease defined as reported history of asthma, rhinitis or eczema in the child’s biological mother or father (no vs. yes). Indicators of socioeconomic status in this setting were whether the mother (no vs. yes) or father (no vs. yes) of the participant were working, and whether the child was living with both parents (no vs. yes).

#### Other sources of air pollution

The following variables were considered as additional sources of exposure: current cigarette exposure at home (no vs. yes), type of heating used at home (other vs. coal and gas), pavement of the nearest street (no vs. yes) (“no” indicates the nearest street was mainly a dirt road), the child’s preferred play area (inside vs outside), and time spent at home (less than 3 h/day vs. 3–6 h/day, more than 6 h/day).

### Exposure assessment

In the absence of ambient measurements of emission or of personal exposures, we used the proximity to the open pit mines as proxy of exposure [25, 26]. We used the residential proximity to the gold and the copper mines, and the average distance to both. During the fieldwork, the geographical coordinates of each place of residence was obtained using global positioning system (GPS). The locations of the main extraction places were geocoded using official information given by the companies. The Harvesine formula for distance was used to account for the curvature of the earth [27]. The lower quartile of distance to each mine separately and of the average distance was selected a-priori as cut-off point of the exposure categories.

### Statistical analysis

To assess associations between mining exposure and health outcomes, we conducted a three-step analysis 1) Logistic regressions were fitted for the two outcomes using the dichotomized proximity variable; 2) Bayesian structured additive regressions were used to estimate a possible non-linear effect on the respiratory diseases related to the proximity to the mines; results of this part were used to calibrate the subsequent modeling step; 3) Bayesian models with a parametric distance function were used to estimate associations between distance to the mines and the outcomes, and to identify a minimum distance to the point sources beyond which the health impact is considered non statistically significant. To adjust for possible regional effects, which are not captured by distance to the mine or the individual’s characteristics, we included an additional spatial effect. All analyses where done using the statistical software R [28].

#### Logistic regression

We estimated associations between distance from the mines (i.e., gold mine, copper mine and average distance to both mines) and the respiratory diseases using logistic regression models adjusting for potential confounders. Distance was dichotomized at the first quartile, i.e., the quartile closest to the point sources was categorized as 1 for children living in the closest quartile, 0 otherwise. A variable was considered a potential confounder if it was associated with the outcome in the crude models. All subsequent analyses were adjusted for these variables. To assess the potential influence of item non-response, we performed sensitivity analyses employing multiple imputation (MI) under the assumption of missing at random. Seven data sets were generated with the MI procedure and combined to create pooled estimates using Rubin’s rules [29]. We then compared the pooled with the complete case estimates. The MI process and model estimation were done using the R libraries Amelia [30] and Zelig [31], respectively.

#### Bayesian structured additive regression

We investigated non-linear associations between air pollution exposure proxies (i.e., the distance variable) and respiratory diseases using Bayesian generalized structured additive regression (STAR) [32] from a Bayesian perspective. The model has the equation: *logit*(*π*_*i*_) = *β*_0_ + *f*(*d*_*ik*_) + Xβ, where *f*(*d*_*ik*_) is an unknown smooth function of the distance *d*_*ik*_ of child *i* to mine *k*. X comprises additional confounders with effects β. We used Bayesian P-splines *f*(*d*_*ik*_). We assumed independent diffuse priors for the estimated effects of the potential confounders β, the intercept *β*_0_ and the P-splines [33].

The Markov chain Monte Carlo methods was implemented through the software BayesX [34] using 70,000 iterations including a burn-in phase of 15,000 iterations. Two chains were generated with different starting values. Finally, convergence was evaluated with trace plots and the convergence statistics provided by the software BayesX.

We used the results of the Bayesian Structured Additive Regression to calibrate the prior distributions for the Bayesian models with parametric distance functions. This approach is preferable when little or nothing is known about the shape of the relationship between the proximity to the point sources of pollution and the disease [35]. Then, STAR models could be seen as a calibration step for models with a parametric form for the distance.

#### Bayesian models with a parametric distance function

We fitted Bayesian models with a parametric distance function proposed by [25, 36, 37] and used the Bayesian perspective suggested by [26], and extended these models for multiple diseases with one point sources and a spatial effect. These models have the function [38],

1 $$ logit\left({\pi}_i(s)\right)={\beta}_0+f\left({d}_{ik}\left|{\alpha}_k,{\phi}_k,{\delta}_k\right.\right)+\mathrm{X}\upbeta +\mathrm{S}\left(\mathrm{s}\right) $$

where *f*(.) is the parametric function of the distance proposed in [39] as

2 $$ f\left({d}_{ik}\left|{\alpha}_k,{\phi}_k,{\delta}_k\right.\right)=\left\{\begin{array}{rr}\hfill 1+{\alpha}_k,& \hfill if\ {d}_{ik}\le\ {\delta}_k\\ {}\hfill 1+{\alpha}_k \exp \left\{{\left(\frac{d_{ik}-{\delta}_k}{\phi_k}\right)}^2\right\},& \hfill if\ {d}_{ik}>{\delta}_k\end{array}\right. $$

Here 1 + *α*_*k*_ reflecting the excess of odds ratio at the mine, *δ*_*k*_ represents the radius of the plateau around the mine, *ϕ*_*k*_ represents the rate at which the risk decreases with each additional kilometer of distance to the edge of the plateau. An additional spatial effect S(s) corrects for any regional differences in the risk of the disease, which could not be explained by the potential confounders or by the proximity to the mines. Model construction was performed in a hierarchical procedure:

- Model 0: *logit*(*π*_*i*_) = *β*_0_ + Xβ, including only potential confounders
- Model 1: *logit*(*π*_*i*_(*s*)) = *β*_0_ + Xβ + *S*(*s*), including a spatial variation.
- Model 2: *logit*(*π*_*i*_(*s*)) = *β*_0_ + *f*(*d*_*ik*_| *α*_*k*_, *ϕ*_*k*_, *δ*_*k*_) + Xβ, including the parametric proximity function.
- Model 3: *logit*(*π*_*i*_(*s*)) = *β*_0_ + *f*(*d*_*ik*_|*α*_*k*_, *ϕ*_*k*_, *δ*_*k*_) + Xβ + S(s), including an additional spatial effect to explore if there is a remaining spatial variation which could not be explained by the proximity or the confounders.

The models were compared using the deviance information criterion (DIC) [40] where lower values indicate an improvement of the fit. These models were estimated using the software JAGS [41] called from R.

## Results

The final sample included 275 children; 3 children with missing residence location, and 10 children who lived in isolated zones outside of the municipality (thus were outliers) were excluded. Children’s mean (± sd) age was 9.05 years (aged 6 to 15 years), and 46 % were girls. Most children lived with both parents (67 %) and 25 % of mothers worked. More than half of the children reported to be at home 6 h or more per day (54 %). The main place for play was outside (Table 1).

**Table 1:** Sociodemographic characteristics of the study participants for the total population and by different outcomes

	Total^a^	Asthma		Rhinoc.	
	*N* = 275	(*n* = 66; NA^b^)		(*n* = 66; NA^b^)	
Potential confounders	%	n	%	n	%
Sex					
Female	46	28	22	44	35
Male	54	38	26	49	33
Age					
6–7 years	27	14	19	25	34
8–9 years	31	24	28	25	29
10–11 year	30	17	21	31	38
≥ 12 years	12	11	33	12	36
Living with both parents (NA = 9)					
No	29	28	35	29	36
Yes	67	38	21	62	34
Parental atopic disease (NA = 30)					
No	58	27	17	43	27
Yes	31	32	38	43	51
Mother working (NA = 14)					
No	69	54	27	63	33
Yes	25	11	16	26	38
Father working (NA = 22)					
No	8	7	32	6	27
Yes	84	51	22	81	35
Hours child stay at home (NA = 61)					
< 3 h	7	5	25	5	25
3-6 h	17	11	24	18	39
> 6 h	54	44	30	58	39
Place child most of the time (NA = 9)					
Inside	38	22	21	33	31
Outside	59	43	27	58	36
Smoking in child’s presence (NA = 28)					
No	65	44	24	63	35
Yes	24	19	28	24	36
Paved street near to house (NA = 10)					
No	21	18	32	19	33
Yes	76	48	23	74	36
Type of heater (NA = 69)					
Other	29	21	26	29	36
Coal and gas	46	34	27	41	33
Gold mine distance					
1st quartile	25	22	32	28	41
2nd quartile	25	12	17	20	29
3rd quartile	26	20	28	22	31
4th quartile	24	12	18	23	35
Copper mine distance					
1st quartile	25	21	30	31	45
2nd quartile	25	14	20	21	30
3rd quartile	25	14	20	20	29
4th quartile	25	17	25	21	31
Average distance to both mines					
1st quartile	25	24	34	31	44
2nd quartile	25	11	16	21	31
3rd quartile	25	15	22	19	28
4th quartile	25	16	24	22	32

*Abbreviations*: *Rhinoc.* rhinoconjunctivitis, *NA* Missing values

^a^ Percentages include the missing values

^b^ Indicates number of participants without any information regarding to the respective disease under study

The prevalence of asthma was 24 %, and the prevalence of rhinoconjunctivitis was 34 %. The mean (± sd) distance to the gold mine was 2.1 (± 0.26) km (median: 2.1 km; range 1.3–2.8 km) and 1.9 (± 0.37) km (median: 2.0 km; range 0.9–3.1 km) for the copper mine. For the average distance to both mines the mean was 2.1 km (± 0.26) km (median: 2.0; range 1.3–2.7). The prevalence of the diseases among the 25 % of children living closest to the mines, was between 30 and 45 %, and was slightly higher than for the remaining sample (Table 1). A geographical map of children’s place of residence is presented in the Additional file 1: Figure SA.1.

The adjusted odds ratios for the respiratory outcomes were higher with closer proximity to the mines. Strongest associations were estimated for the combined outcome for children living on average closer than 1.8 km to both mines. Asthma was associated with parental history of atopic diseases, living with both parents, and having a working mother (Additional file 1: Figure SB.1). Including these covariates in a multiple regression, did not change the estimated effects of proximity to the mines and respiratory diseases appreciably. Including imputed data did also not change our findings appreciably (Table 2), therefore we used complete case data for the further analyses. The associations estimated using the multiple imputation dataset are presented in Additional file 1: Figure SC.1.

**Table 2:** Association between distance to the mines and respiratory diseases. Sensitivity analysis for missing data. Complete cases, multiple imputation and adjustment for potential confounders

Respiratory disease		Gold mine		Copper mine		Average distance	
		OR^c^	95 % CI	OR^c^	95 % CI	OR^c^	95 % CI
Asthma	Complete cases	1.67	(0.90–3.12)	1.47	(0.79–2.74)	1.62	(0.87–3.00)
	Multiple imputation	1.63	(0.91–2.93)	1.46	(0.78–2.73)	1.75	(0.96–2.99)
	Adjusted multiple imputation^a^	1.71	(0.89–3.28)	1.33	(0.69–2.55)	1.62	(0.82–3.18)
Rhinoc.	Complete cases	1.54	(0.87–2.75)	1.79	(1.02–3.16)	1.72	(1.00–3.00)
	Multiple imputation	1.54	(0.87–2.74)	1.84	(1.03–3.26)	1.72	(0.98–3.00)
	Adjusted multiple imputation^b^	1.56	(0.87–2.79)	1.78	(0.98–3.26)	1.87	(1.05–3.35)

*Abbreviations*: *CI* confidence interval, *OR* odds ratio, *Rhinoc.* rhinoconjunctivitis

^a^ Adjusted for parental atopic diseases, if the child lived with both parents and if the child’s mother worked using logistic regression models

^b^ Adjusted for parental atopic diseases using the logistic regression models

^c^ Reference categories is to live to a distance above of the cut-off points (first quartile) using the logistic regression models

**Fig. 1:**
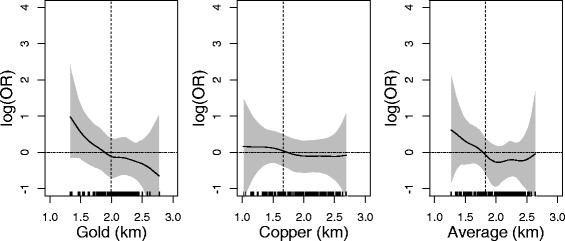
Association between proximity to the mines and asthma adjusted for parental congenital atopic diseases, if the child lived with both parents and if the child’s mother worked. Dotted *vertical line* indicates the first quartile of the distance. Plots are based on participants’ data living within minimum and maximum of the distances from the mines. The shaded area is the 95 % Bayesian confidence intervals

**Fig. 2:**
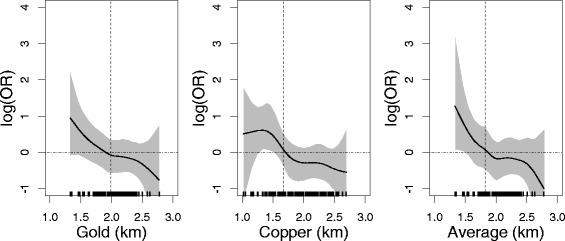
Adjusted associations between proximity to mines and rhinoconjunctivitis for parental congenital atopic diseases. Dotted *vertical line* indicates the first quartile of the distance. Only for subject within distance range from the mine. The shaded area is 95 % Bayesian confidence interval

The adjusted STAR models confirmed the results of the logistic regressions for all outcomes (Figs. 1 and 2) with those living closer to the mines having higher risks for respiratory diseases. A slight “U” shape in the estimations of the associations was seen for the copper mine, and for average distance in both outcomes (Figs. 1 and 2). For the association between distances to the copper mine and asthma no decrease towards zero was estimated. The findings for the two outcomes were consistent, i.e., with closer distance to the gold mine the risk of all respiratory diseases increased about *exp*(1) with confidence interval reaching from *exp*(0) to *exp*(2). Within a distance of 2 km to the gold mine we estimated a positive effect suggesting an increased risk for the respiratory outcomes. Around the copper mine, the risk was increased by *exp*(0.5) with BCIs between *exp*(1) and *exp*(2). The odds ratios were positive up to a distance of around 1.7 km. Combining the proximity to both mines by using the average distance resulted in similar estimates as the analyses of the distance to the gold mine only. The unadjusted models did not differ appreciably from the adjusted models (Additional file 1: Figures SD.1 to SD.2).

Using the STAR models (Figs. 1 to 2), we found a maximum OR of *exp*(1) at the minimum distance to the mines, with the lower and upper limits of the confidence intervals around *exp*(1) and *exp*(2), respectively. The construction of the prior distributions is presented in the Additional file 1: (Section F.1).

The parametric Bayesian models 0 to 3 indicated that model 3, including the proximity-risk function together with the spatial terms resulted in a better fit for all outcomes and exposures (Additional file 1: Table SD.1); the DIC was lower than for other models.

**Fig. 3:**
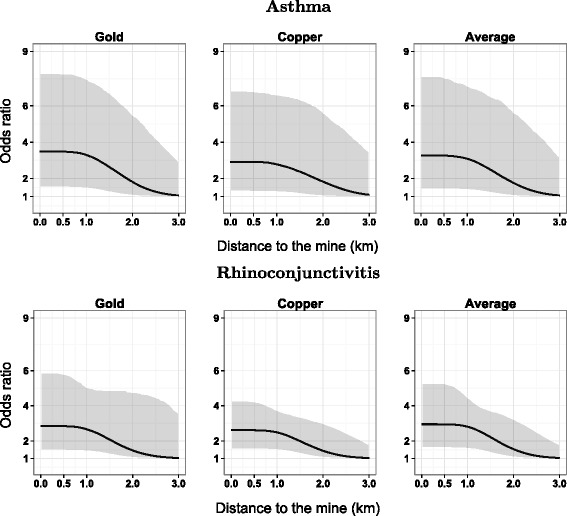
Association between proximity to mines and each respiratory disease using the Model 3. **Gold** mine, **Copper** mine and **Average** distance. The shaded area is 95 % Bayesian confidence interval

Table 3 shows the parameter estimates and the 95 % Bayesian confidence intervals for each mine. Generally, the point estimates of $\widehat{\alpha_k}$ lay within 1.6–2.4, for $\widehat{\phi_k}$ had values of 1.05–1.13 and $\widehat{\delta_k}$ between 0.72 and 0.95. Figure 3 shows the posterior $\widehat{f_k}$ estimates for distance functions. These graphs present similar values at the source of around 2, and it maintains the risk constant until around 1 km of distance from the mines; at further distance, the risk declines to 1 at around 2.5 km of distance to the mine. The 95 % confidence intervals for $\widehat{f_k}$ are above 1 up to a distance of 1.8 km. Plots of the random terms *Ŝ*_*k*_(*s*) using *T* = 40 spatial nodes are presented in the Additional file 1: Figures SE.1 to SE.2.

**Table 3:** Posterior estimates and 95 % Bayesian confidence intervals for parameters of the proximity-odds function, *α*, *ϕ* and *δ* in the model 3 for the three respiratory diseases

Respiratory disease	Mine	$\widehat{\boldsymbol{\alpha}}$ 95%BCI	$\widehat{\boldsymbol{\Phi}}$ 95 % BCI	$\widehat{\boldsymbol{\updelta}}$ 95 % BCI
Asthma	Gold	2.48 (0.51–6.80)	1.08 (0.80–1.46)	0.80 (0.31–1.88)
	Copper	1.92 (0.29–5.81)	1.13 (0.82–1.53)	0.95 (0.31–2.13)
	Average distance	2.26 (0.42–6.62)	1.10 (0.80–1.50)	0.86 (0.31–1.95)
Rhinoc.	Gold	1.85 (0.46–4.87)	1.08 (0.78–1.48)	0.78 (0.28–1.97)
	Copper	1.61 (0.52–3.27)	1.07 (0.81–1.47)	0.78 (0.29–1.67)
	Average distance	1.65 (0.61–4.28)	1.07 (0.80–1.46)	0.77 (0.30–1.59)

*Abbreviations*: *BCI* Bayesian confidence interval, *Rhinoc.* rhinoconjunctivitis

## Discussion

The findings of our community based study in Chile suggest that proximity of children’s residence to open-pit mines, one of the main income sources in many low and middle income countries, is associated with allergic rhinoconjunctivitis and asthma with markedly increased risks in children living closest to the mines. We used different measures of proximity as proxy for ambient air pollution exposure which resulted in comparable results.

The results of our study corroborate previous study from Latin America suggesting increased risks for asthma within a distance of two kilometers to different air pollution sources including power plants, quarries or petroleum refineries [18]. Using categorical distance measures, investigators also found an increased prevalence of respiratory diseases and allergies in children who lived near wood industries in Alaska [19]. Indeed, the use of cut-off points is arbitrary, making the use of STAR models an alternative allowing a broader perspective on the exposure-outcome association in this kind of analysis.

We also estimated the spatial effect to capture the residual variation not accounted for by the population at risk, and to estimate risks across the study area. Models including an effect of distance from the mine on the respiratory diseases resulted in a better fit; however, the improvements were small.

Variables included as potential confounders for asthma were parental history of atopic disease, child’s mother working and living with both parents. For rhinitis only parental history of atopic disease played a role and was included in the adjusted models. Exposure to indoor sources of pollutants such as type of heating and smoking in presence of the child was not relevant for the the respiratory diseases in our data. Parental occupation was not included, because the work environment did not involve exposure to contaminants that could be associated with asthma attacks or any other respiratory disease in the children. We considered residential area rather than school location, as children reported to spend most leisure time at home. We assumed children spent only the remaining time at school. Since our study population only consisted of children from two different schools, the variation of school-based exposure will be small.

Our study has several limitations. Asthma and rhinoconjunctivitis diagnosis was reported by parents or children themselves even though the questionnaire was intended to be answered by the children’s parents; this could have led to an inaccurate report of the prevalence of the diseases, because most parents or children may not have been aware of the diagnosis. However, the inclusion of asthma medication data should have improved the accuracy of the outcome assessment. Likewise, the symptoms of rhinoconjunctivitis were self-reported. However, both questions were previously validated within the ISAAC study [24].

There were no emission measurements available of the mining related exposures. Unfortunately, we do not have any information on the particle concentration or composition in the mining community. In general, the particles include minerals, chemicals, heavy metals, aluminum, mercury, and arsenic; several of these components are known to be associated with impaired respiratory health [42]. Moreover, it is also known that particulate material may act as allergenic compound and thus might not only contribute to non allergic asthma and rhinitis, but also to allergic and atopic asthma [43]. Additionally, we did not incorporate information on wind direction or other geographical information (i.e. the angle of the mines to each residence) [44]. Since these data were not available in our study region, we used spatial models to estimate risks. However, misclassification of exposure is likely non-differential which may bias our findings likely towards the null. However, this approach can be used for risk estimation in other communities in Latin America with similar mining procedures where measurement data are often not available.

We found wide Bayesian confidence intervals which may be explained by the “flat” likelihood of these kinds of models, and the high standard errors in the estimates that usually result in posterior estimates close to the mean of the prior distribution [38, 45], especially for the *α*_*k*_. Therefore, we used different types of prior distribution to evaluate this choice of the *α* parameter (see Section F.2 and Additional file 1: Figures SF.1 to SF.3) as a sensitivity analysis. Our findings of two previous studies [45, 46] that suggested prior distributions for parametric form in the distance function should be carefully chosen.

This study has several strengths. We assessed the models specifications by permitting distance-risk relationships to be nonlinear and free of a pre-specified shape. We compared traditional statistical methods with most recent approaches proposed in the literature as easy to interpret. Semiparametric methods suffer from a number of limitations for incorporating information about a realistic shape of the proximity-risk [26, 35]. Therefore, we combined Bayesian semiparametric models to specify the prior distributions for parametric form, since no or little epidemiological data on the association was available.

We implemented complex models in standard statistical software. These implementations could be extended to consider a model for multiple diseases and multiple sources, avoiding the specification of a single model for each disease, potentially taking into account scenarios in which the outcomes are correlated.

## Conclusions

Our findings suggest that proximity to mines using open-pit procedures increases the risk for asthma and rhinoconjunctivitis in children. In communities with high concentrations of environmental pollution from mining facilities, the adverse effect on children’s health highlights the importance of stricter emissions regulating policies. Most important in terms of public health would be emission control and reduction at the emission source. In addition, education campaigns regarding the identification and control of respiratory disease in order to improve the diagnosis and treatment of respiratory diseases in children are suggested. Finally, the proposed threshold distance might be used as an initial indicator to define zones which should be restricted for residential use. Such a policy might implicate the relocation of parts of the population. Adequate prediction models would allow estimations of possible health impacts of such a policy in terms children’s health in the affected areas. Impacts of such a policy should also be examined from different societal perspectives.

## Abbreviations

BCI, Bayesian confidence interval; CI, confidence interval; GAM, Generalized additive models; GPS, Global positioning system; ISAAC, International study of asthma and allergies in childhood; Rhinoc., Rhinoconjunctivitis

## Additional file

Additional file 1: Proximity to mining industries and respiratory diseases in children of a Northern Chilean Community: A Cross-sectional study Supplemental Material. (PDF 3236 kb)
