# Supplementary material for: Proximity to mining industry and respiratory diseases in children in a community in Northern Chile: A cross-sectional study
Source: Environ Health. 2016 Jun 7;15:66. doi: 10.1186/s12940-016-0149-5 (PMC4897925; doi:10.1186/s12940-016-0149-5)
Supplement: Additional file 1: — Proximity to mining industries and respiratory diseases in children of a Northern Chilean Community: A Cross-sectional study Supplemental Material. (PDF 3236 kb) [file 12940_2016_149_MOESM1_ESM.pdf]

RESEARCH

Proximity to Mining Industries and Respiratory Diseases in Children of a Northern Chilean Community: A Cross-sectional study Supplemental Material

Ronald Herrera[1,4*], Katja Radon[1], Ondine S. von Ehrenstein[2], Stella Cifuentes[1], Daniel Moraga Muñoz[3] and Ursula Berger[4]

*Correspondence: rherrera@med.lmu.de
[1]Occupational and Environmental Epidemiology and NetTeaching Unit, Institute for Occupational, Social and Environmental Medicine University Hospital Munich (LMU), Ziemssenstr. 1,80930, Munich,Germany
Full list of author information is available at the end of the article

A Geographical position of children's houses

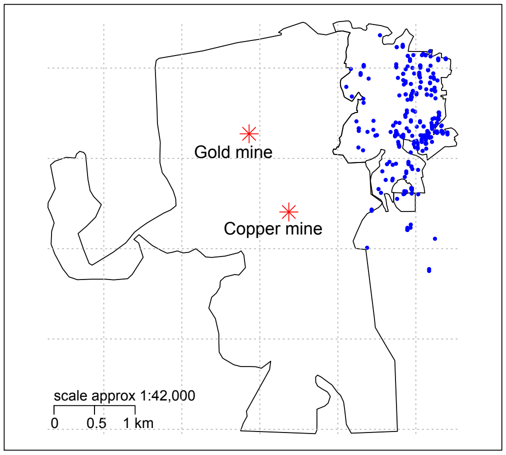

Figure A.1. Geographical position of point sources and child's residence. Original positions were changed using jitter in order to keep confidentiality.

B Unadjusted pre imputation Odds Ratios (OR) for association between sociodemographic factors and respiratory diseases

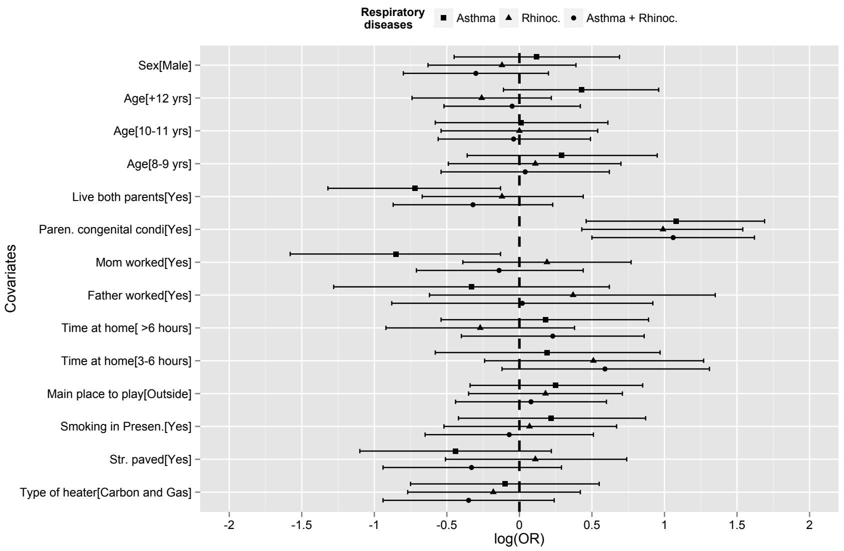

Figure B.1. Unadjusted pre imputation OR for association between sociodemographic factors and respiratory diseases. Bars represent the 95% confidence interval. **Rhinoc.= Rhinoconjunctivitis**

C Multiple imputation process

Given the presence of missing data (Table 1), seven data set were generated and the Rubin's rules [1] were used to calculate the parameter estimates. Results of the multiple imputation process are given in Figure C.1. Figure shows the unadjusted Odds Ratios (OR) for each covariate after the imputation process, these were calculated using logistic regression models.

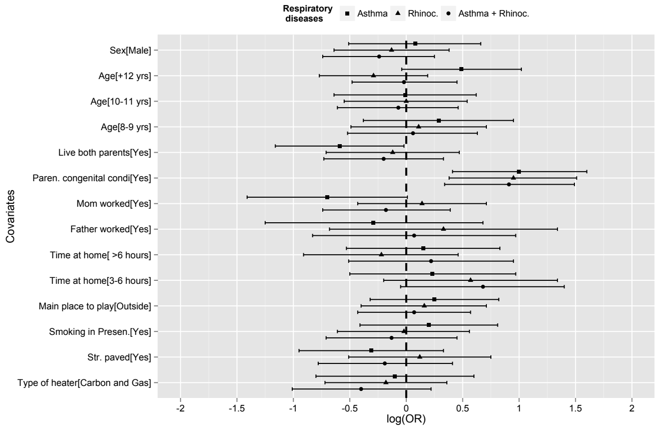

Figure C.1. Unadjusted post imputation OR for association between sociodemographic factors and respiratory diseases. Bars represents the 95% confidence interval.

D Unadjusted STAR models

The models estimated were as follows:

$$logit(\pi_i) = \beta_0 + f(d_{ik}) \tag{1}$$

The resulting estimation are presented in Figures D.1 to D.2

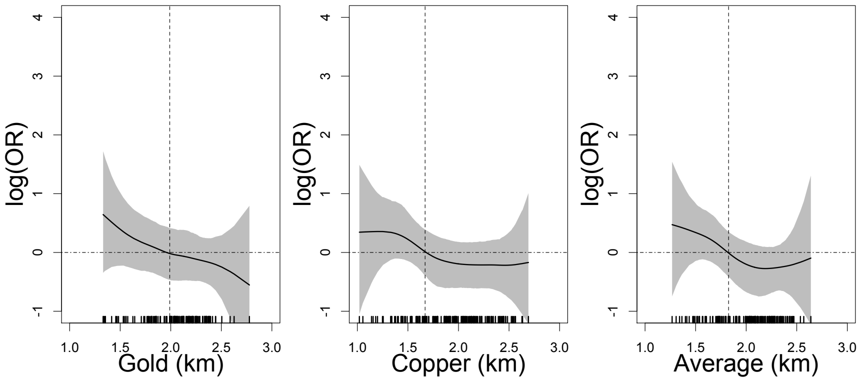

Figure D.1. Effect of the proximity to the mines on **asthma** unadjusted.
Dotted vertical line indicates the first quartile for the distance.
Only for subjects who live within distance range from the mine. Shaded area is 95% Bayesian confidence interval.

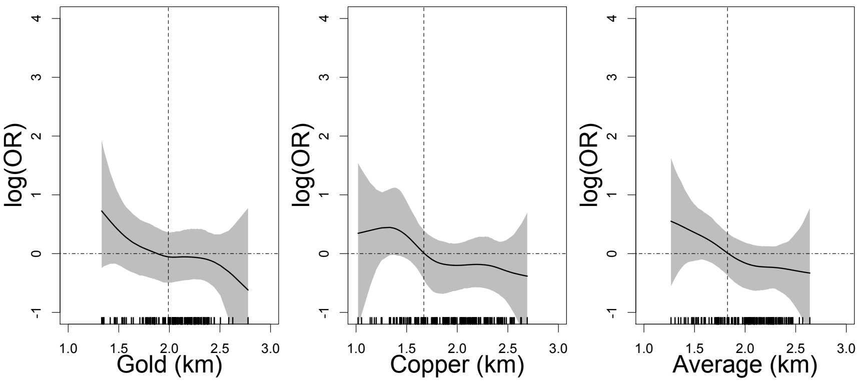

Figure D.2. Effect of the proximity to mines on **rhinoconjunctivitis** unadjusted. Dotted vertical line indicates the first quartile for the distance.
Only for subjects who live within distance range from the mine. Shaded area is 95% Bayesian confidence interval.

Table D.1. Comparison of alternative model specification for the proposed models.

Respiratory disease	Model	Gold mine DIC (pD)	Copper mine DIC (pD)	Average distance DIC (pD)
Asthma	**0**	227.53 (4.02)	227.53 (4.02)	227.53 (4.02)
	**1**	227.20 (4.09)	227.20 (4.09)	227.20 (4.09)
	**2**	226.29 (4.77)	229.41 (5.35)	227.49 (5.31)
	**3**	225.75 (4.19)	227.84 (4.32)	226.56 (4.15)
Rhinoc.	**0**	300.80 (1.99)	300.80 (1.99)	300.80 (1.99)
	**1**	300.04 (1.93)	300.04 (1.93)	300.04 (1.93)
	**2**	299.70 (2.92)	297.95 (3.05)	297.59 (2.74)
	**3**	298.82 (2.74)	297.19 (2.89)	296.85 (2.65)

Abbreviations: DIC, deviance information criterion; pD, effective number of estimated parameters; Rhinoc., rhinoconjunctivitis

E Spatial effect for Model 3

The random term $\mathbf{S}(\mathbf{s})$ represents a residual spatial component in the models and is independent from the presence of the mines. It reflects all the variation not accounted by the covariates and the distance function. Different approaches are used to model this term, depending the context, perhaps, Gaussian random field in the analysis of lattice data [2]. However, [3, 4] and [5, 6] (in the context of modelling of the association between risk and relation of putative source) used an approach with a thin-plate splines interpolate, this last approach was used in this paper to model this effect, this semi-parametric term is favourable because of the computational advantages.

For the spatial compound specification, given a set of $\mathbf{T}$ spatial nodes of the N children locations, $\mathbf{S}(\mathbf{s})$ has a low-rank representation following the form:

$$\mathbf{S}(\mathbf{s}) = \mathbf{Z}(\mathbf{s})\mathbf{b} \tag{2}$$

with $\mathbf{b}$ is a T-dimensional vector of random coefficients to control of the spatial smoothing, $\mathbf{Z}(\mathbf{s})$ is the *sth* row of the design matrix

$$\mathbf{Z} = \mathbf{Z_T}\boldsymbol{\Omega}_\mathbf{T}^{-\mathbf{1/2}} \tag{3}$$

In 3, $\mathbf{Z_T}$ and Ω_T are the spatial correlation matrix between the N children residential locations and the T nodes and that among the nodes, respectively. Both matrices are based in a isotropic spatial correlation function in the radial basis function, it means we used $C(r) = r^2 \log(r)$ where r is the Euclidean distance, resulting in $\mathbf{Z_T} = [C\{d(s,t)\}]$ and $\Omega_T = [C\{d(t,t')\}]$, with $t, t' = 1, \ldots, t_T$, completing the thin-spline representation necessary in 2.

Finally, priors distributions for the thin plate approach in Equation 2 are as follows:

$$\mathbf{b} \sim N(\mathbf{0_T}, \tau_b \mathbf{I_T}) \tag{4}$$

And $\mathbf{0_T}$ and $\tau_b\mathbf{I_T}$ are T-dimensional null vectors and identity matrix. The hyperparameter τ_b is a precision parameter of the spatial residual component, making $\tau_b \sim Gamma(0.001, 0.001)$ does not affect the posterior estimates [3], but it could cause slow convergence and mixture problems in Markov chain Monte Carlo (MCMC) algorithms, to avoid this inconvenient, we used $\mathbf{b} \sim N(\mathbf{0_T}, \tau_b\mathbf{I_T})$, with $\tau_b \sim IGamma(9, 3)$, this approach was suggested by Dreassi et al.[7]

We used $T = 40$ nodes were chosen using **clara** algorithms within **R** library **SemiPar**. (Put Reference)

E.0.1 Spatial effects estimated

Predicted effects for **b** using the Model 3.

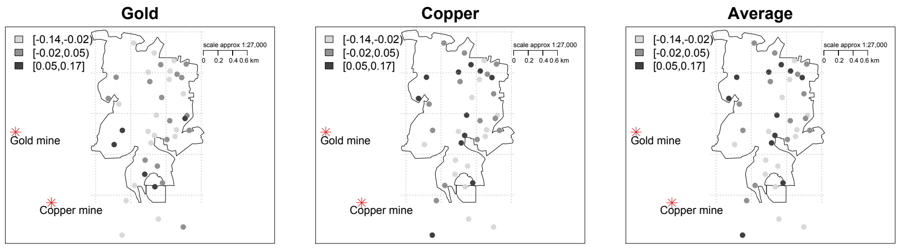

Figure E.1. Spatial effects predicted, $\hat{\mathbf{S}}(\mathbf{s})$ low-rank Gaussian random field for **asthma** and distance to gold mine (left), copper mine (center) and average distance (right).

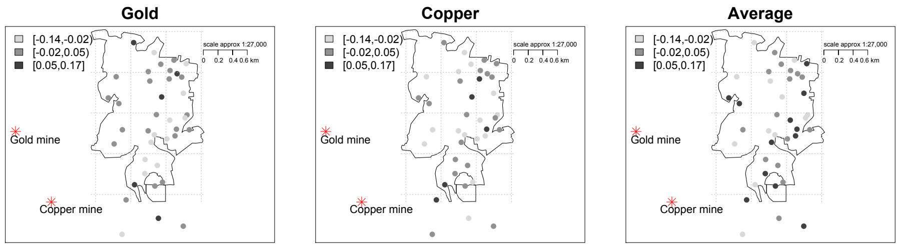

Figure E.2. Spatial effect predicted, $\hat{\mathbf{S}}(\mathbf{s})$ low-rank Gaussian random field for **rhinoconjunctivitis** and distance to gold mine (left), copper mine (center) and average distance (right).

F Prior construction for the Bayesian parametric models

F.1 Prior elections

Election of the prior distributions have been study previously [2, 5], suggesting special care in the priors' elections specially for α_k and ϕ_k. We followed the proposal in Li et al.[8], doing a one-to-one transformation of the parameters $(u_k, v_k) = (log(1 + \alpha_k), log(\phi_k))$.

We considered mutually independent prior Normal distributions on (u_k, v_k),

$$log(1 + \alpha_k) = u_k \sim N(\mu_{u_k}, \sigma^2_{u_k}) \quad (5)$$

$$log(\phi_k) = v_k \sim N(\mu_{v_k}, \sigma^2_{v_k}) \quad (6)$$

For α_k and ϕ_k. A simple transformation allows to calculate μ_{u_k} for a proposed value of the mean of α_k, μ_α, which is obtained from the STAR models. The hyperparameters μ_{u_k} and $\sigma^2_{u_k}$ for α_k specify a distribution reflecting the point estimate and uncertainty obtained with the STAR models at the nearest distance. For ϕ_k, μ_{v_k} and $\sigma^2_{v_k}$ reflected the decrease of the risk over distances from the edge of the plateau at which the risk had decreased.(33). The parameter δ_k is distributed as a $Gamma(\kappa_1, \kappa_2)$. The hyperparameters κ_1 and κ_2 defined an informative prior reflecting the radius of the plateau that no observation at distances below than 0.87 km. The spatial effect $\mathbf{S}(\mathbf{s})$ collected the residual variation across the region not accounted for the potential confounders or by the proximity to the mines. $\mathbf{S}(\mathbf{s})$ was estimated using Bayesian thin-plate splines.

F.2 Sensitivity analysis for the parameter α

As sensitivity analysis of the prior specification on the parameter α, posterior densities were obtained and compared from different choices of prior distributions for the Model 3 using asthma as outcome. The priors were defined as:

- $\sigma^2_{\alpha_k} = 1.5$
- $\sigma^2_{\alpha_k} = 2$
- $\alpha_k \sim Unif(-1, \exp(1))$
- $\alpha_k \sim Unif(-1, \exp(1.5))$
- $\alpha_k \sim Unif(-1, \exp(2))$

Figures F.1 to F.3 show the posterior distributions in the Model 3 using different priors distributions.

Models are conditioned to the election of a good prior distribution when the election is a uniform distribution, however the election of normal distribution on parameters transformation should be showed more stability. The election of prior flat or non informative distribution must be considerer to no use in the uniform case because the modes are sensitive to the election of the upper limit, it was also mentioned by other authors [2, 7, 8]. The parametrization proposed by Li et al. showed more robustness in our study. Posterior densities using others outcomes presented a similar behaviour (not shown).

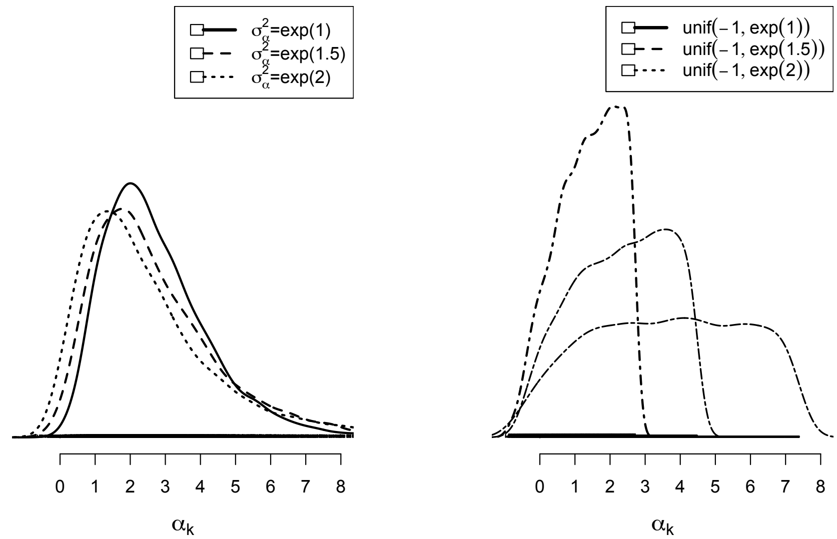

Figure F.1. Posterior density plots for α parameter for distance to the **gold mine** and **asthma** in the Model 3 using normal priors on $\log(\mathbf{1} + \alpha_{\mathbf{k}})$ (left) and uniform distributions on α_k (right)

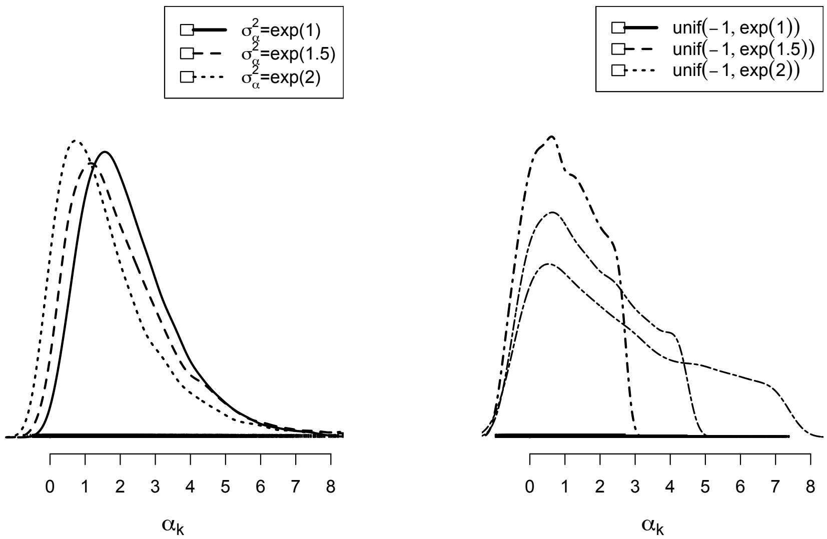

Figure F.2. Posterior density plots for α parameter for distance to the **copper mine** and **asthma** in the Model 3 using normal priors on $\log(\mathbf{1}+\alpha_\mathbf{k})$ (left) and uniform distributions on α_k (right)

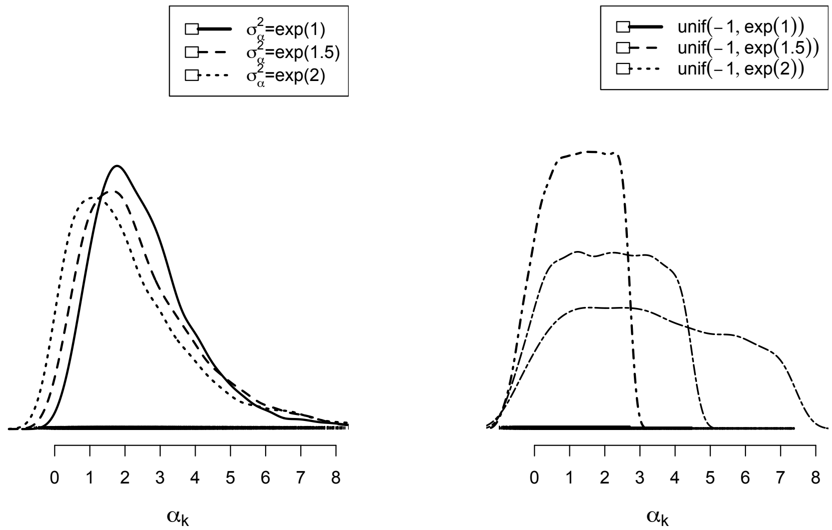

Figure F.3. Posterior density plots for α parameter for distance to the **average distance to the mines** and **asthma** in the Model 3 using normal priors on $\log(\mathbf{1}+\alpha_{\mathbf{k}})$ (left) and uniform distributions on α_k (right)

Author details
[1]Occupational and Environmental Epidemiology and NetTeaching Unit, Institute for Occupational, Social and Environmental Medicine University Hospital Munich (LMU), Ziemssenstr. 1,80930, Munich,Germany. [2]Department of Community Health Sciences Fielding School of Public Health University of California Los Angeles, USA, 650 Charles E. Young Dr. South, Los Angeles, USA. [3]Universidad Iberoamericana de Ciencias y Tecnología. Past staff of Facultad de Medicina, Latin American Center of the Center for International Health LMU, Universidad Católica del Norte Coquimbo, Padre Miguel de Olivares 1620, Santiago, Chile. [4]Institute for Medical Informatics, Biometry and Epidemiology-IBE University of Munich (LMU), Marchioninistr. 15, Munich, Germay.

References
1. Little R, Rubin D. Statistical Analysis with Missing Data. Wiley Series in Probability and Statistics; 1987.
2. Wakefield JC, Morris SE. The Bayesian Modelling of Disease Risk in Relation to a Point Source. Journal of the American Statistical Association. 2001;96(453):77–91.
3. Crainiceanu C, Ruppert D, Wand MP. Bayesian Analysis for Penalized Spline Regression Using WinBUGS. Journal of Statistical Software. 2005;14(14):1–24.
4. Crainiceanu C, Ruppert D, Carroll R, Joshi A, Goodner B. Spatially Adaptative Bayesian Penalized Splines with Heteroscedastic Errors. Journal of Computational and Graphical Statistics. 2007;16(2):265–288.
5. Calculli C, Pollice A, Serinelli M. Spatial analysis of the risk of multiple cancers in relation to a petrochemical plant. Environmetrics. 2011;23(2):175–182.
6. Rodrigues A, Diggle P, Assuncao R. Semiparametric approach to point source modelling in epidemiology and criminology. Journal of the Royal Statistical Society: Series C (Applied Statistics). 2010;59(Part 3):533–542.
7. Dreassi E, Lagazio C, Maule M, Magnani C, Biggeri A. Sensitivity analysis of the relationship between disease occurrence and distance from a putative source of pollution. Geospatial Health. 2008;2(2):263–271.
8. Li S, Mukherjee B, Batterman S. Point source modeling of matched case-control data with multiple disease subtypes. Statistics in Medicine. 2012;31:3617–3637.
